# Supplementary material for: Mapping the evolution of fertility support policies in China: A content and instrumental analysis
Source: PLoS One. 2025 Oct 9;20(10):e0332137. doi: 10.1371/journal.pone.0332137 (PMC12510515; doi:10.1371/journal.pone.0332137)
Supplement: S1 Appendix — (ZIP) [file pone.0332137.s001.zip › S1 Appendix. 226 original policy documents/82-国务院关于印发国家人口发展规划(2016—2030年)的通知(FBM-CLI-2-289464).doc]

国务院关于印发国家人口发展规划(2016—2030年)的通知

**发布部门：** [国务院](https://www.pkulaw.com/law/chl?Aggs.IssueDepartment=5)

**发文字号：**国发〔2016〕87号

**发布日期：**2016.12.30

**实施日期：**2016.12.30

**时效性：** [现行有效](https://www.pkulaw.com/law/chl?Aggs.TimelinessDic=01)

**效力级别：** [国务院规范性文件](https://www.pkulaw.com/law/chl?Aggs.EffectivenessDic=XC0203)

**法规类别：** [人口计划管理](https://www.pkulaw.com/law/chl?Aggs.Category=09701)

国务院关于印发国家人口发展规划（2016－2030年）的通知
（国发〔2016〕87号）

各省、自治区、直辖市人民政府，国务院各部委、各直属机构：
　　现将《国家人口发展规划（2016－2030年）》印发给你们，请认真贯彻执行。

　　国务院
2016年12月30日

　　国家人口发展规划（2016－2030年）

　　人口问题始终是人类社会共同面对的基础性、全局性和战略性问题。为积极有效应对我国人口趋势性变化及其对经济社会发展产生的深刻影响，促进人口长期均衡发展，根据《[中华人民共和国人口与计划生育法](https://www.pkulaw.com/chl/18c6c388fc14eaa9bdfb.html)》、《[中华人民共和国国民经济和社会发展第十三个五年规划纲要](https://www.pkulaw.com/chl/3fd7b48d6cb8d951bdfb.html)》和《[中共中央 国务院关于实施全面两孩政策改革完善计划生育服务管理的决定](https://www.pkulaw.com/chl/8564756bae805261bdfb.html)》精神，特编制本规划。本规划旨在阐明规划期内国家人口发展的总体要求、主要目标、战略导向和工作任务，是指导今后15年全国人口发展的纲领性文件，是全面做好人口和计划生育工作的重要依据，并为经济社会发展宏观决策提供支撑。

　第一章　规划背景

－－人口发展的关键转折期

　　进入21世纪后，我国人口发展的内在动力和外部条件发生了显著改变，出现重要转折性变化。准确把握人口变化趋势性特征，深刻认识这些变化对人口安全和经济社会发展带来的挑战，对于谋划好人口长期发展具有重大意义。

　第一节　人口现状

　　我国全面推行计划生育40多年来，人口过快增长得到有效控制，人口再生产类型实现历史性转变，对资源环境的压力有效缓解，有力促进了经济发展、社会进步和民生改善，为现代化建设提供了重要保障和基础性支撑，为全面建成小康社会奠定了坚实基础。“十二五”时期，根据我国人口发展变化趋势，党中央、国务院审时度势作出调整完善生育政策的重大决策部署，深化计划生育服务管理改革，人口和计划生育工作取得了新成效。
　　－－人口总量平稳增长。“十二五”期间年均自然增长率保持在5‰左右，2015年末总人口为13.75亿人。
　　－－人口结构不断变化。出生人口性别比连续下降至113.51，60岁及以上老年人口占比达到16.1%，15－59岁劳动年龄人口于2011年达到峰值后持续下降，家庭户均人口规模减少。
　　－－人口素质稳步提升。人均预期寿命提高到76.34岁。孕产妇死亡率和婴儿死亡率分别降至20.1/10万和8.1‰，提前实现联合国千年发展目标。劳动年龄人口平均受教育年限达到10.23年。
　　－－人口城乡结构发生重大变化。常住人口城镇化率从2010年的49.95%提升至2015年的56.1%，流动人口从2.21亿人增加到2.47亿人。
　　－－重点人群保障水平不断提高。2015年农村贫困人口为5575万人，较2010年减少了66.3%。老年人、残疾人等群体社会保障体系和公共服务体系逐步健全。家庭发展能力得到增强。

　第二节　发展态势

　　根据联合国《世界人口展望：2015年修订版》预测，今后较长时期内世界人口将保持上升趋势，人口总量将从2015年的73亿上升到2030年的85亿，2050年接近100亿，发展中国家人口占比继续上升，中国人口占比持续下降。世界多数国家已经或正在步入老龄化社会，中国老龄化水平及增长速度将明显高于世界平均水平。
　　我国人口发展既符合世界一般性规律，又具有自身特点，今后15年人口变动的主要趋势是：
　　－－人口总规模增长惯性减弱，2030年前后达到峰值。实施全面两孩政策后，“十三五”时期出生人口有所增多，“十四五”以后受育龄妇女数量减少及人口老龄化带来的死亡率上升影响，人口增长势能减弱。总人口将在2030年前后达到峰值，此后持续下降。
　　－－劳动年龄人口波动下降，劳动力老化程度加重。劳动年龄人口在“十三五”后期出现短暂小幅回升后，2021－2030年间将以较快速度减少。劳动年龄人口趋于老化，到2030年，45－59岁大龄劳动力占比将达到36%左右。
　　－－老龄化程度不断加深，少儿比重呈下降趋势。“十三五”时期，60岁及以上老年人口平稳增长，2021－2030年增长速度将明显加快，到2030年占比将达到25%左右，其中80岁及以上高龄老年人口总量不断增加。0－14岁少儿人口占比下降，到2030年降至17%左右。
　　－－人口流动仍然活跃，人口集聚进一步增强。预计2016－2030年，农村向城镇累计转移人口约2亿人，转移势头有所减弱，城镇化水平持续提高。以“瑷珲－腾冲线”为界的全国人口分布基本格局保持不变，但人口将持续向沿江、沿海、铁路沿线地区聚集，城市群人口集聚度加大。
　　－－出生人口性别比逐渐回归正常，家庭呈现多样化趋势。伴随经济社会发展以及生育政策调整完善等，出生人口性别比呈稳步下降态势。核心家庭（由已婚夫妇及其未婚子女组成的家庭）和直系家庭（由父母同一个已婚子女及其配偶、子女组成的家庭）是主要的家庭形式，单人家庭、单亲家庭以及“丁克家庭”的比例将逐步提高。
　　－－少数民族人口增加，地区间人口变化不平衡。2015年我国少数民族人口总量为1.17亿，占比8.5％，少数民族生育率高于全国平均水平，人口比例还将进一步提高。在一些民族地区各民族人口发展不均衡，一些边境地区青壮年人口流失比较严重。

　第三节　问题和挑战

　　今后15年我国人口发展进入深度转型阶段，人口自身的安全以及人口与经济、社会等外部系统关系的平衡都将面临不可忽视的问题和挑战。
　　－－实现适度生育水平压力较大。我国生育率已较长时期处于更替水平以下，虽然实施全面两孩政策后生育率有望出现短期回升，但受生育行为选择变化等因素影响，从长期看生育水平存在走低的风险。
　　－－老龄化加速的不利影响加大。人口老龄化加快会明显加大社会保障和公共服务压力，凸显劳动力有效供给约束，人口红利减弱，持续影响社会活力、创新动力和经济潜在增长率。
　　－－人口合理有序流动仍面临体制机制障碍。城乡、区域间人口流动仍面临户籍、财政、土地等改革不到位形成的制度性约束，人口集聚与产业集聚不同步、公共服务资源配置与常住人口不衔接、人口城镇化滞后于土地城镇化等问题依然突出，不利于有效支撑国家重大区域战略实施。
　　－－人口与资源环境承载能力始终处于紧平衡状态。本世纪中叶前我国人口总量将保持在13亿以上，人口对粮食供给的压力持续存在，人口与水资源短缺的矛盾始终突出，人口与能源消费的平衡关系十分紧张。边境地区人口安全问题需高度关注。
　　－－家庭发展和社会稳定的隐患不断积聚。小型化和空巢化家庭抗风险能力低，养老抚幼、疾病照料、精神慰藉等问题日益突出。出生人口性别比长期失衡积累的社会风险不容忽视。
　　总的看，未来15年特别是2021－2030年，我国人口发展进入关键转折期，但人口众多的基本国情不会根本改变，人口对经济社会发展的压力不会根本改变，人口与资源环境的紧张关系不会根本改变。既面临诸多问题和潜在风险挑战，同时继续存在劳动力总量充裕、仍处于人口红利期等有利条件，统筹解决人口问题有较大的回旋空间。完善人口发展战略和政策体系，促进人口长期均衡发展，最大限度地发挥人口对经济社会发展的能动作用，对全面建成小康社会、实现中华民族伟大复兴的中国梦，具有重大现实意义和深远历史意义。

　第二章　总体思路

－－实施人口均衡发展国家战略

　　面对人口发展重大趋势性变化，必须把人口均衡发展作为重大国家战略，加强统筹谋划，把握人口发展的有利因素，积极有效应对风险挑战，努力实现人口自身均衡发展，并与经济社会、资源环境协调发展。

　第一节　总体要求

　　高举中国特色社会主义伟大旗帜，以马克思列宁主义、毛泽东思想、邓小平理论、“三个代表”重要思想、科学发展观为指导，全面贯彻党的十八大和十八届三中、四中、五中、六中全会精神，深入贯彻习近平总书记系列重要讲话精神，认真落实党中央、国务院决策部署，紧紧围绕统筹推进“五位一体”总体布局和协调推进“四个全面”战略布局，牢固树立和贯彻落实创新、协调、绿色、开放、共享的发展理念，以促进人口均衡发展为主线，立足战略统筹，强化人口发展的战略地位和基础作用，坚持计划生育基本国策，鼓励按政策生育，充分发挥全面两孩政策效应，综合施策，创造有利于发展的人口总量势能、结构红利和素质资本叠加优势，促进人口与经济社会、资源环境协调可持续发展，为全面建成小康社会、实现中华民族伟大复兴的中国梦提供坚实基础和持久动力。
　　要坚持以下原则：
　　－－坚持综合决策。切实将人口融入经济社会政策，在经济社会发展战略规划计划、经济结构战略性调整、投资项目和生产力布局、城乡区域关系协调、可持续发展等重大决策中，充分考虑人口因素，不断健全人口与发展综合决策机制。
　　－－突出以人为本。坚持以人民为中心的发展思想，优先投资于人的全面发展，建立健全面向全人群、覆盖全生命周期的人口政策体系，促进共同参与、共享发展，增强人民群众获得感和幸福感。
　　－－强化正向调节。尊重人口规律，顺应经济社会发展要求和群众根本利益，完善服务保障政策，将生育水平调控并维持在适度区间，推动人口结构优化调整、人口素质不断提升、人口流动更加有序，持续增强人口资源禀赋。
　　－－加强风险防范。加强超前谋划和战略预判，重视把握人口各要素之间，以及人口与经济社会、资源环境等外部要素间的相互关系，提早防范和综合应对潜在的人口系统内安全问题和系统间的安全挑战，切实保障人口安全。
　　－－深化改革创新。积极转变人口调控理念和方法，统筹推进生育政策、计划生育服务管理制度、家庭发展支持体系和治理机制综合改革，完善人口预测预报预警机制，健全重大决策人口影响评估制度。

　第二节　主要目标

　　到2020年，全面两孩政策效应充分发挥，生育水平适度提高，人口素质不断改善，结构逐步优化，分布更加合理。到2030年，人口自身均衡发展的态势基本形成，人口与经济社会、资源环境的协调程度进一步提高。
　　－－人口总量。总和生育率逐步提升并稳定在适度水平，2020年全国总人口达到14.2亿人左右，2030年达到14.5亿人左右。
　　－－人口结构。出生人口性别比趋于正常，性别结构持续改善。劳动力资源保持有效供给，人口红利持续释放。
　　－－人口素质。出生缺陷得到有效防控，人口健康水平和人均预期寿命持续提高，劳动年龄人口平均受教育年限进一步增加，人才队伍不断壮大。
　　－－人口分布。常住人口城镇化率稳步提升，户籍人口城镇化率加快提高，主要城市群集聚人口能力增强。人口流动合理有序，人口分布与区域发展、主体功能布局、城市群发展、产业集聚的协调度达到更高水平。
　　－－重点人群。民生保障体系更加健全，老年人、妇女、儿童、残疾人、贫困人口等群体的基本权益得到有效保障，生活水平持续提高，共建共享能力明显增强。

| 专栏1　预期发展目标 | | | | | |
| --- | --- | --- | --- | --- | --- |
| 领　域 | 主要指标 | 单位 | 2015年 | 2020年 | 2030年 |
| 人口总量 | 全国总人口 | 亿人 | 13.75 | 14.2 | 14.5 |
|  | 总和生育率 |  | 1.5-1.6 | 1.8 | 1.8 |
| 人口结构 | 出生人口性别比 |  | 113.5 | ≤112 | 107 |
| 人口素质 | 人均预期寿命 | 岁 | 76.3 | 77.3 | 79 |
|  | 劳动年龄人口平均受教育年限 | 年 | 10.23 | 10.8 | 11.8 |
| 人口分布 | 常住人口城镇化率 | % | 56.1 | 60 | 70 |

　第三节　战略导向

　　实现人口发展目标，必须从经济社会全局高度和国家中长期发展层面谋划人口工作，深入实施国家人口均衡发展战略，明确并贯彻以下战略导向：
　　－－注重人口内部各要素相均衡。推动人口发展从控制人口数量为主向调控总量、优化结构和提升素质并举转变。推进全面两孩政策实施，平缓人口总量变动态势，避免人口达到峰值后快速下降，发挥政策最大效应。加强出生人口性别比治理，促进社会性别平等。切实提高出生人口素质，努力挖掘各年龄段人口潜能，推动人口红利向人才红利转变。
　　－－注重人口与经济发展相互动。准确把握经济发展对人口变动的影响，综合施策缓解经济因素带来的生育率下降等人口发展问题。统筹城乡区域协调发展，统筹技术、产业、公共服务、就业同步扩散，引导人口与经济布局有效对接。充分发挥人口能动作用，为经济增长提供有效人力资本支撑和内需支撑。实施积极的老龄化政策，防范和化解对经济增长的不利影响。
　　－－注重人口与社会发展相协调。完善国家基本公共服务制度体系，推动基本公共服务常住人口全覆盖，有序推进农业转移人口市民化。着力补齐重点人群发展短板，构建多层次养老服务体系，保障妇女儿童、残疾人合法权益，实施贫困人口精准脱贫，促进社会公平正义。尊重个人和家庭在人口发展中的主体地位，坚持权利义务对等，推动人口工作由主要依靠政府力量向政府、社会和公民多元共治转变。
　　－－注重人口与资源环境相适应。根据不同主体功能区定位要求，健全差别化的人口政策，多措并举引导人口向优化开发和重点开发区域适度集聚，支持鼓励限制开发和禁止开发区域的人口自愿迁出，严格控制超大、特大城市人口规模。加大环境治理与保护力度，可持续开发利用自然资源，推动形成绿色发展方式和生活方式，着力增强人口承载能力。

　第三章　推动实现适度生育水平

－－延续人口总量势能优势

　　适度生育水平是维持人口良性再生产的重要前提。要针对人口变动态势，做好超前谋划和政策储备，健全生育服务和家庭发展支持体系，引导生育水平提升并稳定在适度区间，保持和发挥人口总量势能优势，促进人口自身均衡发展。

　第一节　健全生育政策调控机制

　　坚持计划生育基本国策，实施全面两孩政策。坚持党政一把手亲自抓、负总责，落实和完善计划生育目标管理责任制。改革完善计划生育服务管理，实行生育登记服务，优化服务流程，推行网上办事，进一步简政便民。密切关注生育水平过高和过低地区人口发展态势，加强分类指导，因地制宜、综合施策，积极发挥计生协会等社会组织作用，引导群众负责任、有计划、按政策生育。积极推进实施各民族平等的计划生育政策，促进同区域内不同民族的均衡发展。科学评估经济增长和社会发展对生育行为的影响，做好全面两孩政策效果跟踪评估，密切监测生育水平变动态势，做好政策储备，完善计划生育政策。

　第二节　合理配置公共服务资源

　　健全妇幼健康计划生育服务体系，提升妇幼健康和计划生育服务能力。实施妇幼健康计划生育服务保障工程，通过增加供给、优化结构、挖掘潜力，强化孕产妇和新生儿危急重症救治能力建设，进一步降低孕产妇和婴儿死亡率。做好优生优育全程服务，为妇女儿童提供优质的孕前优生健康检查、住院分娩、母婴保健、避孕节育、儿童预防接种等服务，做好流动孕产妇和儿童跨地区保健服务以及避孕节育的接续。加强出生缺陷综合防治，开展出生缺陷发生机理和防治技术研究，推进新生儿疾病筛查、诊断和治疗工作。加强妇幼保健计划生育服务管理能力建设。
　　加强科学预测，合理规划配置儿童照料、学前和中小学教育、社会保障等资源，满足新增公共服务需求。引导和鼓励社会力量举办非营利性妇女儿童医院、普惠性托儿所和幼儿园等服务机构。鼓励和推广社区或邻里开展幼儿照顾的志愿服务。推进生育保险和基本医疗保险合并实施，确保职工生育期间的生育保险待遇不变。在大型公共场所、公共交通工具、旅游景区景点等设置母婴室或婴儿护理台，保障母婴权益。

专栏2　妇幼健康计划生育服务保障工程

向孕产妇提供覆盖生育全过程的基本医疗保健服务，实行生育登记服务制度，加强出生人口监测预测，继续实施免费孕前优生健康检查，落实国家规定的计划生育基本技术服务。继续实施扩大国家免疫规划，按规定提供孕产期保健和儿童保健服务。扩大农村妇女“两癌”（宫颈癌、乳腺癌）检查项目覆盖范围。全面改善妇幼健康服务条件，支持省、市、县三级妇幼健康服务机构能力建设，提升孕产妇和新生儿危急重症救治能力。2020年前力争新增产床8.9万张，增加产科医生和助产士14万名。

　第三节　完善家庭发展支持体系

　　建立完善包括生育支持、幼儿养育、青少年发展、老人赡养、病残照料、善后服务等在内的家庭发展政策。完善税收、抚育、教育、社会保障、住房等政策，减轻生养子女家庭负担。完善计划生育奖励假制度和配偶陪产假制度。鼓励雇主为孕期和哺乳期妇女提供灵活的工作时间安排及必要的便利条件。支持妇女生育后重返工作岗位。增强社区幼儿照料、托老日间照料和居家养老等服务功能。完善殡葬基本公共服务。加强家庭信息采集和管理，为家庭发展政策的制定和实施提供依据。大力发展家庭服务业。加强婚姻家庭辅导，推进新型家庭文化建设，开展幸福家庭创建活动。
　　加大对计划生育家庭的扶助力度，对全面两孩政策实施前的独生子女家庭和农村计划生育双女家庭，继续实行现行各项奖励扶助政策，在社会保障、集体收益分配、就业创业、新农村建设等方面予以倾斜。完善计划生育家庭特别扶助制度，加大对残疾人家庭、贫困家庭、计划生育特殊家庭、老年空巢家庭、单亲家庭等的帮扶支持力度，充分发挥社会工作服务机构和社会工作者的专业作用。

　第四章　增加劳动力有效供给

－－注重人口与经济良性互动

　　综合应对劳动年龄人口总量下降和结构老化趋势，全面提升劳动力质量，挖掘劳动力供给潜能，加强与就业政策和劳动力市场建设的有机衔接，为经济社会发展提供有效人力资本支撑。

　第一节　提升新增劳动力质量

　　努力适应经济向形态更高级、分工更精细、结构更合理演进，发展方式向依靠持续的知识积累、技术进步、劳动力素质提升转变，着力培养具有国际竞争力的创新型、应用型、高技能、高素质大中专毕业生和技能劳动者，提高新增劳动力供给质量。加快完善国民教育体系，不断提高基本公共教育服务均等化水平，2020年普及高中阶段教育，迈入高等教育普及化门槛（毛入学率达到50%），2030年达到更高普及水平，劳动年龄人口平均受教育年限进一步提升。全面提高教育质量，切实提升大中专毕业生就业创业能力。优化人才培养机制，促进人才培养链与产业链、创新链有机衔接。深入推进协同育人，深化产教融合、校企合作，大力培养应用型人才和技术技能人才。全面提升高校创新人才培养能力，建设好一批世界一流大学和一流学科，深化高校创新创业教育改革，增强毕业生社会责任感、创新精神和实践能力。建设面向人人的就业创业服务平台。实施高校毕业生就业创业促进计划，建立涵盖毕业生校内校外各阶段、求职就业各环节、创新创业全过程的服务体系。

　第二节　挖掘劳动者工作潜能

　　提升就业能力，推动农村劳动力转型。面向现代农业发展，构建有效的新型职业农民培育制度体系，加快推动传统农民成为新型职业农民，建立高素质现代农业生产经营者队伍。持续推进农业富余劳动力进城务工并稳定生活，落实农业转移人口就业扶持政策，实施新生代农民工职业技能提升计划，健全职业培训、就业服务、劳动维权“三位一体”的工作机制。凝聚政府与市场合力，优化环境并健全支持政策，建设一批返乡创业园区和县乡特色产业带，为外出务工人员返乡创业创造条件。
　　促进劳动者人力资本积累。通过全方位投资人力资本，充分发挥劳动者工作潜能。大力发展继续教育，强化企业在职工培训中的主体作用，完善以就业技能、岗位技能提升和创业为主的培训体系，持续提升企业职工劳动技能和工作效能。提升劳动者健康素质，全面开展职业健康服务，落实职业健康检查制度，加强职业病防治。强化职业劳动安全教育。支持大龄劳动力就业创业，加强大龄劳动力职业培训，提高就业技能和市场竞争力，避免其过早退出就业市场。

专栏3　大龄劳动力人力资本开发行动

适应我国老龄化发展趋势，积极借鉴国际有益经验，通过教育培训、健康服务、就业促进等方式鼓励大龄失业人员回归劳动力市场。探索建立养老金长缴多得的激励约束机制；加强大龄劳动力在岗继续教育培训，落实完善职业培训补贴、职业技能鉴定补贴等政策，支持大龄劳动力提升就业技能；加强职业健康服务，提高大龄劳动力健康水平；落实税收优惠、社会保险补贴、创业担保贷款等扶持政策，鼓励各类企业吸纳大龄失业人员就业；加强公共就业服务网络平台建设，为大龄失业人员提供更多个性化职业指导、职业介绍、政策咨询等公共就业服务；结合大龄失业人员特点，提供更多非全职就业、志愿服务和社区工作等岗位。

　第三节　积极开发老年人力资源

　　充分发挥老年人参与经济社会活动的主观能动性和积极作用。实施渐进式延迟退休年龄政策，逐步完善职工退休年龄政策，有效挖掘开发老年人力资源。大力发展老年教育培训。鼓励专业技术领域人才延长工作年限，积极发挥其在科学研究、学术交流和咨询服务等方面的作用。鼓励老年人积极参与家庭发展、互助养老、社区治理、社会公益等活动，继续发挥余热并实现个人价值。

　第四节　有效利用国际人才资源

　　树立人力资源开发利用全球视野，实施更积极、更开放、更有效的国际人才培养和引进政策。制定并完善出入境和长期居留、税收、保险、住房、就医、子女入学、配偶安置等配套措施，加快推进简化“绿卡”办理手续，大力吸引海外高层次人才回国或来华创新创业。完善外国人永久居留制度，放宽技术技能型人才取得永久居留资格的条件，探索实行技术移民并逐步形成完善有效的政策体系。支持外国人才申报和参与国家级科研项目，更加严格有效保护知识产权，维护创造发明人的合法权益。发展国际人才市场，培育一批国际人才中介服务机构。结合国家“一带一路”建设战略实施和国际产能合作，鼓励有条件的企业、高校、科研院所“走出去”培养和吸引使用当地优秀人才。积极开展高水平的国际合作办学，创新人才国际联合培养模式。开发国（境）外优质教育培训资源，完善出国（境）培训管理制度和措施，进一步提高出国（境）培训质量。

　第五章　优化人口空间布局

－－促进人口与资源环境永续共生

　　推动城乡人口协调发展，完善以城市群为主体形态的人口空间布局，促进人口分布与国家区域发展战略相适应，引导人口有序流动和合理分布，实现人口与资源环境永续共生。

　第一节　持续推进人口城镇化

　　加快推进以人为核心的城镇化，引导人口流动的合理预期，畅通落户渠道，到2020年实现1亿左右农业转移人口和其他常住人口在城镇落户，全面提高城镇化质量。按照尊重意愿、自主选择、因地制宜、分步推进、存量优先、带动增量的原则，区分超大、特大和大中小城市以及建制镇，实施差别化落户政策，促进有能力在城镇稳定就业和生活的农业转移人口举家进城落户。将具备条件的县和特大镇有序设置为市，增加中小城市数量，优化大中城市市辖区规模和结构，拓展农业转移人口就近城镇化空间。

专栏4　推动1亿非户籍人口在城市落户方案

2016－2020年，户籍人口城镇化率年均提高1个百分点以上，年均转户1300万人以上。

进一步拓宽落户通道。除极少数超大城市外，全面放宽农业转移人口落户条件。调整完善超大城市和特大城市落户政策，区分城市的主城区、郊区、新区等区域，分类制定落户政策，重点解决符合条件的普通劳动者落户问题。调整完善大中城市落户政策，大中城市均不得采取购买房屋、投资纳税等方式设置落户限制。

制定实施配套政策。加大对农业转移人口市民化的财政支持力度并建立动态调整机制，建立财政性建设资金对吸纳农业转移人口较多城市基础设施投资的补助机制，建立城镇建设用地增加规模与吸纳农业转移人口落户数量挂钩机制。顺应农民保留土地承包权、流转土地经营权的意愿，实行所有权、承包权、经营权分置并行，探索建立进城落户农民土地承包权、宅基地使用权、集体收益分配权维护和自愿有偿退出机制。完善城镇基本公共服务政策，确保城市新老居民同城同待遇。

　第二节　推动城市群人口集聚

　　以城市群为主体形态促进大中小城市和小城镇协调发展，优化提升东部地区城市群，培育发展中西部地区城市群，推动人口合理集聚。对京津冀、长三角、珠三角等城市群，要严格控制超大城市和特大城市人口规模，有序引导人口向中小城市集聚。对长江中游、成渝地区等城市群，要进一步做大做强中心城市，加强对周边欠发达地区的辐射带动作用，打造全国重要的人口集聚区。对山东半岛、海峡西岸、辽中南等东部地区城市群，要进一步加强区域内大中小城市联动发展，增强对中西部转移人口的吸引力。对哈长、中原、关中、北部湾、山西中部、呼包鄂榆、黔中、滇中、兰西、宁夏沿黄、天山北坡等城市群，要加快形成更多支撑区域发展的增长极，引导区域内人口就近集聚。力争今后15年上述19个城市群集聚的常住人口占全国比重稳步提升，特别是城镇人口总量占比增幅更高。

　第三节　改善人口资源环境紧平衡

　　制定和完善与主体功能区相配套的人口政策。要统筹考虑国家战略意图和区域资源禀赋，在开展资源环境承载能力评价的基础上，科学确定不同主体功能区可承载的人口数量，实行差别化人口调节政策。对人居环境不适宜人类常年生活和居住的地区，实施限制人口迁入政策，有序推进生态移民。对人居环境临界适宜的地区，基本稳定人口规模，鼓励人口向重点市镇收缩集聚。对人居环境适宜和资源环境承载力不超载的地区，重视提高人口城镇化质量，培育人口集聚的空间载体，引导产业集聚，增强人口吸纳能力。
　　促进人口绿色发展。实施人口绿色发展计划，积极应对人口与资源环境的紧张矛盾，增强人口承载能力。大力推行创新驱动、资源集约节约、低碳环保的绿色生产方式，推广绿色低碳技术和产品，严格限制高耗能、高污染行业发展，节约集约利用土地、水和能源等资源，促进资源循环利用。积极倡导简约适度、绿色低碳、文明节约的生活方式，推广绿色建筑，鼓励绿色出行。
　　保障边境地区人口安全。要从维护国家安全的高度，多措并举稳住边境人口适度规模，优化人口结构和分布。统筹运用人口发展、产业促进、转移支付、公共服务和社会管理等政策，努力扩大就业、增加边民收入、提高公共服务水平，让边境各族群众安居乐业。加强人口跨境流动管理，促进边境地区繁荣发展。

　第四节　完善人口流动政策体系

　　深化户籍制度改革，切实保障进城落户农业转移人口与城镇居民享有同等权利和义务。全面实施居住证制度，推进居住证制度覆盖全部未落户城镇常住人口，保障居住证持有人享有国家规定的各项基本公共服务和办事便利。鼓励地方各级政府根据本地实际不断扩大对居住证持有人的公共服务范围并提高服务标准。以人口为基本要素，完善公共服务资源配置，使基本公共服务设施布局、供给规模与人口分布、环境交通相适应，增强基本公共服务对人口集聚和吸纳能力的支撑。深化财政制度改革，建立农业转移人口市民化成本分担机制。深化农村集体产权制度改革，探索建立进城落户农民土地承包权、宅基地使用权、集体收益分配权维护和自愿有偿退出机制。研究完善支撑东北地区等老工业基地全面振兴的人口发展政策，适应西部大开发要求鼓励人口向西部地区迁移。健全全国流动人口分布、生存发展状况的动态监测体系，完善流动人口服务管理体制机制。

　第六章　促进重点人群共享发展

－－推动人口与社会和谐共进

　　老年人、妇女、儿童、残疾人和贫困人口，是人口发展中必须特别关注的重点人群。要构建管长远的制度框架，制定有针对性的政策措施，创造条件让重点人群共享发展成果，促进社会和谐与公平正义。

　第一节　积极应对人口老龄化

　　针对人口老龄化程度不断加深的趋势，要加强顶层设计，做到及早应对、科学应对、综合应对。坚持持续、健康、参与、公平的原则，加快构建以社会保障、养老服务、健康支持、宜居环境为核心的应对老龄化制度框架，完善以人口政策、人才开发、就业促进、社会参与为支撑的政策体系。建立更加公平可持续的社会保障制度，加快城乡居民全覆盖，逐步提高基本养老和基本医疗保险统筹层次，确保基金安全可持续运行。大力发展企业年金、职业年金、个人储蓄性养老保险和商业医疗保险，在试点基础上推出个人税收递延型养老保险。探索建立长期护理保险制度，开展长期护理保险试点。全面建立针对经济困难高龄、失能老年人的补贴制度，做好与长期护理保险的衔接。加快完善以居家为基础、社区为依托、机构为补充、医养结合的养老服务体系，增加养老服务和产品供给。建设预防、医疗、康复、护理、安宁疗护等相衔接的覆盖全生命周期的医疗服务体系，强化对老年常见病、慢性病的健康指导和综合干预，提升中医保健、体检体测、体育健身等健康管理水平。完善家庭养老支持措施，建设无障碍的老年友好型社区和城市，营造良好社会氛围，形成敬老、养老、助老的社会风尚。

　第二节　促进妇女全面发展和未成年人保护

　　坚持男女平等基本国策，将性别平等全面纳入法律体系和公共政策，促进融入社会文化，切实保障妇女合法权益，消除性别歧视，提高妇女的社会参与能力和生命健康质量。加强出生人口性别比综合治理，营造男女平等、尊重女性、保护女童的社会氛围，加大打击非医学需要的胎儿性别鉴定和选择性别的人工终止妊娠行为力度。深入开展关爱女孩行动，改善女孩生存环境，建立健全有利于女孩家庭发展的帮扶支持政策体系。
　　坚持儿童优先原则，完善未成年人保护和儿童福利体系。探索适合国情的儿童早期综合发展指导模式，发展适度普惠型儿童福利制度。统筹推进农村留守儿童关爱和困境儿童保障工作，建立未成年人保护响应机制，构建以家庭监护为基础、国家监护为保障、社会监督为补充的保障制度，加强对流浪未成年人的救助保护，完善儿童收养制度。加强儿童健康干预和儿科诊疗能力建设，改善贫困地区儿童营养状况。

　第三节　保障残疾人合法权益

　　增强残疾人制度化保障服务能力，全面实施困难残疾人生活补贴制度和重度残疾人护理补贴制度，建立残疾儿童康复救助制度，有条件的地方对贫困残疾人、重度残疾人基本型康复辅助器具配置和家庭无障碍改造给予补贴。健全残疾人托养照料和康复服务体系，大力开展社区康复，为贫困残疾人和重度残疾人提供基本康复服务。健全残疾人教育体系，对家庭经济困难的残疾儿童实行12年免费教育，对残疾儿童普惠性学前教育予以资助，对有劳动能力和就业意愿的残疾人按规定提供免费就业创业服务。发展残疾人文体事业，推动公共文化体育场所免费或低收费向残疾人开放。加强残疾人友好环境建设，完善城乡无障碍设施，推动信息无障碍发布。发展残疾人慈善事业和服务产业，培育服务残疾人的社会组织和企业，积极引入新的业态和科技成果。

　第四节　实现贫困人口精准脱贫

　　加大脱贫攻坚力度，坚持精准扶贫、精准脱贫的基本方略，通过发展生产、易地搬迁、生态补偿、教育支持、医疗救助、低保兜底等有效措施，确保到2020年我国现行标准下农村贫困人口实现脱贫，贫困县全部摘帽，解决区域性整体贫困。根据经济社会发展水平，完善贫困标准动态调整机制。适应可持续发展新阶段新要求，完善贫困人口精准识别、精准扶持和精准脱贫的长效机制。探索建立符合国情的贫困人口治理体系，推动扶贫开发由主要解决绝对贫困向缓解相对贫困转变，由主要解决农村贫困向统筹解决城乡贫困转变，实现全体人民共同迈入全面小康社会、共同迈向现代化。

　第七章　规划实施

－－完善人口与发展综合决策机制

　　本规划由国务院有关部门和地方各级政府组织实施。各地区、各部门要高度重视、求真务实、开拓创新、攻坚克难，确保规划目标和任务顺利完成。

　第一节　强化人口数据支撑

　　发挥人口基础信息对决策的支撑作用，切实推进人口基础信息共建共享。推动人口健康信息化建设，加强人口基础信息采集和统计工作，加快国家人口基础信息库建设，整合分散在教育、公安、民政、人力资源社会保障、卫生计生、统计等部门的人口数据和信息资源，实现就学升学、户籍管理、婚姻家庭、殡葬事务、就业创业、生育和健康、人口普查和抽样调查等人口基础信息的互联互通、动态更新和综合集成。加强人口数据开发和开放利用，为政府部门、企事业单位、社会公众做好人口信息服务。

　第二节　建立人口预测预报制度

　　结合世界人口预测前沿技术方法，研发适合我国的人口预测技术和模型。在人口普查和抽样调查的基础上，加强人口中长期预测。健全人口动态监测和评估体系，科学监测和评估人口变动情况及趋势影响。建立常态化的人口预测预报机制，定期发布国家人口预测报告。推进分区域的人口预测预报工作。

　第三节　开展重大决策人口影响评估

　　以现有人口计划为基础，完善年度人口发展形势会商机制，监测评估人口变动情况及趋势影响，提出重大人口发展政策建议。科学预测和分析人口因素对重大决策、重大改革和重大工程建设的影响，促进相关经济社会政策与人口政策有效衔接。加强与国家高端智库合作，完善重大人口政策咨询机制，提高决策科学合理性。在社会稳定风险评估框架内，试点增加人口风险评估内容，建立重点评价清单。科学评估经济增长和社会发展对人口的影响，加强人口安全风险防控，做好政策预研预案储备。

　第四节　健全规划实施机制

　　加强组织领导。建立健全国家促进人口发展工作协调机制。加强人口战略研究，统筹重大政策研究制定，协调解决人口发展中的重大问题。国家发展改革委要牵头推进规划实施和相关政策落实，监督检查规划实施情况。各有关部门要切实履行职责，根据本规划提出的各项任务和政策措施，研究制定配套政策和具体实施方案，推动相关专项规划与本规划的衔接协调。地方各级政府要全面贯彻落实本规划，编制本地区的人口发展规划，建立健全工作机制，把规划的重点任务落到实处。
　　做好宣传引导。坚持正确的舆论导向，深入开展人口国情、人口规划和人口政策的宣传解读，及时解答社会各界关注的热点问题，主动回应社会关切，合理引导社会预期。充分发挥各类媒体的作用，深入开展群众喜闻乐见的宣传活动，为政策实施营造良好舆论氛围。
　　推进国际合作。加强世界人口发展趋势研究，积极开展人口发展领域国际合作，促进人口老龄化、国际减贫等政策交流与合作，引导人口发展领域国际规则制定。推动落实2030年可持续发展议程涉人口领域目标。对外宣介我国人口政策及计划生育的成就和经验，为我国人口发展营造良好国际环境。
　　开展监测评估。国家发展改革委牵头建立规划实施动态监测、定期通报制度，加强对规划实施情况的跟踪分析，定期组织对规划实施情况的评估，及时发现和解决规划执行过程中存在的突出问题，以5年为期定期组织对本规划实施情况的评估，确保规划各项任务落到实处。

© 北大法宝：（[www.pkulaw.com](https://www.pkulaw.com/)) 专业提供法律信息、法学知识和法律软件领域各类解决方案。北大法宝为您提供丰富的参考资料，正式引用法规条文时请与标准文本核对。
欢迎查看所有[产品和服务](https://www.pkulaw.com/helps/2.html)。法宝快讯：[如何快速找到您需要的检索结果？](https://www.pkulaw.com/helps/70.html)[法宝](https://www.pkulaw.com/helps/69.html)V6有何新特色？

扫描二维码阅读原文

**原文链接：**<https://www.pkulaw.com/chl/81dfb521dd8b807bbdfb.html>
